# Cerebral oxygen delivery in newborns with congenital heart disease by phase contrast MRI

**DOI:** 10.1186/1532-429X-17-S1-M9

**Published:** 2015-02-03

**Authors:** Jessie Mei Lim, Theo Kingdom, Brahmdeep Saini, Susan Blaser, Lars Grosse-Wortmann, Shi-Joon Yoo, Edward J Hickey, Christopher Macgowan, Steven Miller, Mike Seed

**Affiliations:** 1 The Hospital for Sick Children, Mississauga, ON, Canada

## Background

Congenital heart disease (CHD) is associated with in utero brain dysmaturation and abnormal cerebral vasculature [1,2]. Phase contrast (PC) MRI allows non-invasive quantification of cerebral blood flow (CBF) and, combined with arterial oxygen saturation (SaO_2_) and haemoglobin (Hgb) concentration, can measure cerebral oxygen delivery (CDO_2_). We hypothesized that CDO_2_ would be lower in newborns with CHD than normals.

## Methods

We measured CBF and brain volume (BV) in 32 unsedated newborns (21 normals and 11 CHD) as part of an IRB approved study using a 1.5T Siemens Avanto MRI. CBF was measured according to a previously published technique using a single PC acquisition prescribed perpendicular to both internal carotid arteries (ICA) and basilar artery (BA) at the clivus level [3]. We also measured flows in the vertebral arteries (VA), common carotid arteries (CCA) and internal jugular veins (IJV) using a PC acquisition through the neck. Flows were quantified and indexed to BV calculated from a high resolution 3D T2W FSE acquisition. SaO_2_ and Hgb concentrations were measured using conventional blood gases in order to calculate: CDO_2_=SaO_2_×[Hgb]×1.36×CBF [4]. We compared net and indexed CBF and CDO_2_ in the two groups with an unpaired *t*-test with Welch's correction (Table 1). An analysis of CHD subgroups was also performed. The relationship between CBF and gestational age (GA) and agreement between BA vs VA flow sum was examined using Pearson's R.

**Table 1:** Table of mean values for all parameters measured, listed as mean ± SEM. P-values refer to comparisons of each subject group to the control group. The CHD subgroups are as follows: single ventricle physiology (SV), transposition of the great arteries (TGA), coarctation of the aorta (CoA), cyanotic and non-cyanotic CHD.

				Net Value				Indexed to Brain Volume			
Patient Type	Brain Volume (ml)	SaO_2_ (%)	Hgb (g/dL)	CBF (ml/min)	P-Value for CBF	CDO_2_ (ml_O2_/min)	P-Value for CDO_2_	CBF (ml/min/ml_BV_)	P-Value for CBF	CDO_2_ (ml_O2_/min/ml_BV_)	P-Value for CDO_2_

Normal (n=21)	395 ± 12	98	15	138 ± 14		2749 ± 284		0.341 ± 0.027		6.81 ± 0.54	

CHD (n=11)	364 ± 14	90 ± 2.9	15.4 ± 0.44	118 ± 10	0.26	2191 ± 159	0.10	0.321 ± 0.020	0.56	5.99 ± 0.31	0.19

SV (n=4)	364 ± 31	85 ± 6.4	15.5 ± 1.1	122 ± 22	0.58	2101 ± 299	0.15	0.328 ± 0.036	0.78	5.69 ± 0.49	0.15

TGA (n=4)	347 ± 13	89 ± 1.5	15.1 ± 0.38	113 ± 13	0.23	2058 ± 199	0.06	0.329 ± 0.041	0.82	5.97 ± 0.63	0.34

CoA (n=3)	385 ± 28	99 ± 0.67	15.8 ± 0.73	117 ± 20	0.46	2487 ± 382	0.61	0.301 ± 0.032	0.38	6.40 ± 0.59	0.63

Cyanotic (n=7)	365 ± 14	85 ± 2.9	15.7 ± 0.46	124 ± 12	0.47	2198 ± 135	0.09	0.339 ± 0.027	0.96	6.04 ± 0.36	0.24

Non-Cyanotic (n=4)	361 ± 31	100 ± 0.5	15.0 ± 0.98	106 ± 18	0.22	2177 ± 411	0.29	0.290 ± 0.025	0.19	5.89 ± 0.66	0.31

## Results

**Figure 1:**
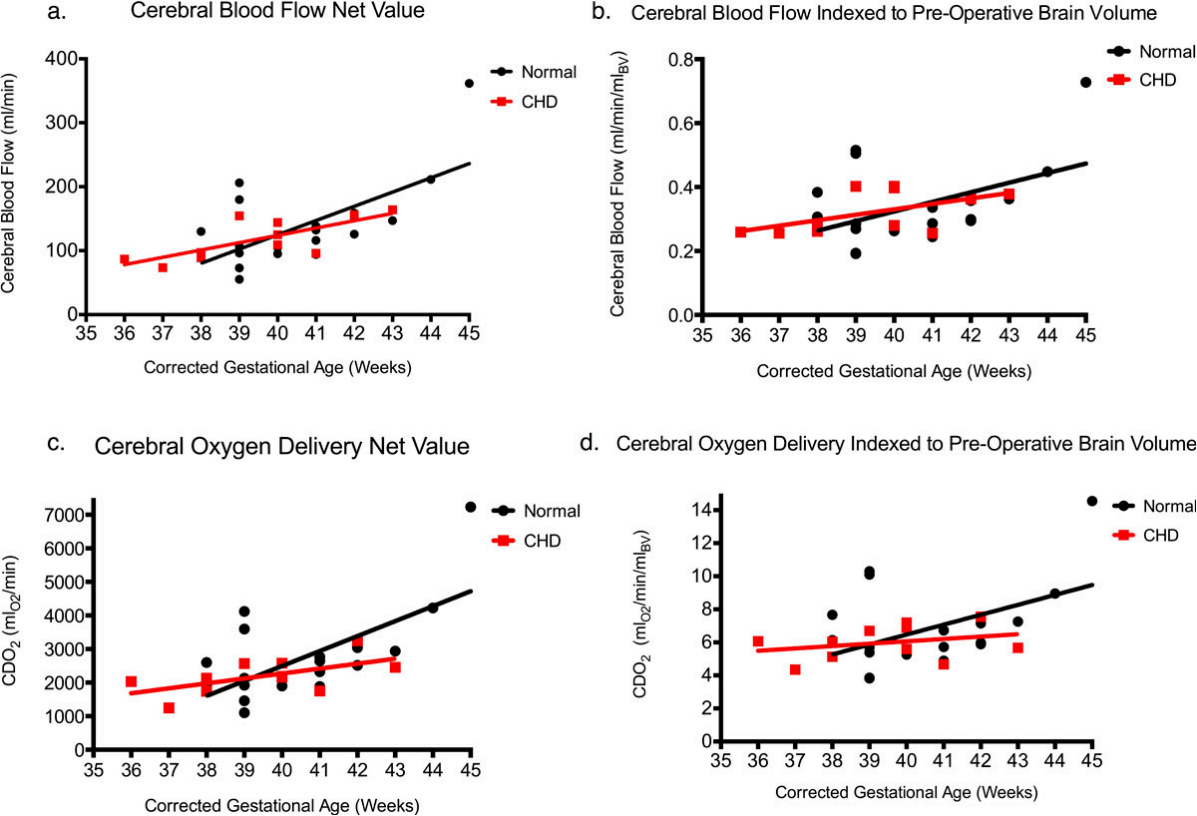
CHD vs control comparisons of parameters plotted against corrected gestational age measured in weeks. a) Net cerebral blood flow (ml/min) vs gestational age; b) Indexed cerebral blood flow to pre-operative brain volume (ml/min/ml_BV_) vs gestational age; c) Net cerebral oxygen delivery (ml_O2_/min) vs gestational age; d) Indexed cerebral oxygen delivery to pre-operative brain volume (ml_O2_/min/ml_BV_) vs gestational age. There is a trend in each parameter towards decreased values in CHD subjects compared to control.

Subjects were scanned at a mean corrected GA of 40 weeks. MRI was performed prior to cardiac surgery in all CHD subjects. The two groups had no difference in GA (p=0.07). There were trends towards lower mean net and indexed CBF in CHD newborns compared with controls (control net CBF 138±14 vs CHD 118±10 ml/min (p=0.26), control indexed CBF 0.341±0.027 vs CHD 0.321±0.020 ml/min/ml_BV_ (p=0.56)) (Table 1, Figure 1a,b). A similar trend in mean net and indexed CDO_2_ was observed in CHD newborns compared with controls (control net CDO_2_ 2749±284 vs CHD 2191±159 ml_O2_/min (p=0.1), control indexed CDO_2_ 6.81±0.54 vs CHD 5.99±0.31 ml_O2_/min/ml_BV_ (p=0.19)) (Figure 1c,d). In keeping with one previous study (3), CBF increased with GA (r=0.7, p=<0.0001). The VA and BA flows were highly correlated (r=0.8, p=0.0002), indicating good accuracy of the PC assessment of CBF. There were no significant differences in measured parameters between subgroups of CHD.

## Conclusions

Although the differences between CBF and CDO_2_ in this small sample of subjects were not statistically significant, there was a clear trend towards lower values for both parameters in CHD subjects. We propose that with a larger sample size, these differences will be significant, in keeping with a demonstrable relationship between cerebral oxygenation and brain maturation in CHD fetuses and newborns.

## Funding

N/A.
